# Is Oral Mucosal Micronucleus Testing an Effective Tool for Biomonitoring Pathology Laboratory Workers Chronically Exposed to Formalin?

**DOI:** 10.30699/ijp.2024.2017360.3224

**Published:** 2024-10-15

**Authors:** Rosana Xavier, Andrea Cristina de Moraes Malinverni, Thiago Guedes Pinto, Fernando Cintra Magalhaes, Daniel Araki Ribeiro

**Affiliations:** 1 *Department of Pathology, Paulista Medical School, Federal University of São Paulo, UNIFESP, Sao Paulo, SP, Brazil*; 2 *Department of Biosciences, Institute of Health and Society, Federal University of São Paulo, UNIFESP, Santos, SP, Brazil*

Dear Editor, 

We have read the recent paper published in the Iranian Journal of Pathology entitled "Formalin Induced Micronucleus Formation in the Buccal Mucosa of Pathology Laboratory Workers"(1). The authors found a significant increase in the percentage of oral micronucleated cells in the pathology laboratory workers when compared to the non-exposed group. Nevertheless, some concepts and guidelines for properly evaluating the paper are presented.

The objectives of the evaluation are based on some guidelines for the use of the micronucleus assay in oral exfoliated cells, as described in detail by the Human Micronucleus Assay Expert Group (2). Of particular importance, it is strongly recommended that a minimum of 2,000 cells per individual be analyzed with a DNA-specific stain in oral mucosal cells. This approach is critical for a high-quality assessment, as such parameters are considered important confounders. In this study, the authors noted that "Papanicolaou staining was used to evaluate the cells containing micronucleus after fixation procedure using the Pathofix spray (PADTAN TEB Co, Tehran, Iran) and drying at room temperature" and "A total of 500 cells were counted for each sample and presence of the cells with micronucleus was reported in percentages" (1). It is important to note that Papanicolaou is not suitable for micronucleus testing in oral mucosal cells because the dye is not reliable for identifying nucleic acids specifically. This is a complicating factor because the identification of micronuclei in this case is very complicated due to the presence of some structures inside the epithelial cells that are equal to micronuclei, such as cytoplasmic granules, leukocytes, or microorganisms (bacteria) (3). This leads to false positives. In addition, the study analyzed only 500 cells per individual. Surely, the total number of cells evaluated is considered very low, considering the results found. 

In the results, the authors were able to present all data in terms of the total number of micronuclei and the percentage of micronucleated cells. Statistical differences were found only for the percentage of micronuclei. How can this be explained? The authors in the manuscript did not elucidate such a discrepancy.

In the Discussion, it was stated that "This discrepancy could be due to the presence of other genotoxic factors, such as air pollution (presence of NO2) and exposure to ionizing radiation, both of which have the potential to increase the frequency of micronucleus. Although these genotoxic factors may influence our results, it was impossible to evaluate them in the current research" (1). This statement does not make sense since the control group was also exposed to air pollution. How is it possible that ionizing radiation can induce genotoxicity in the buccal mucosa (inside the mouth)? 

Finally, it is very important to argue the relationship between cytotoxicity and genotoxicity. It is important to remark that Tolbert *et al.* (4) reported several metanuclear changes indicatory of cytotoxicity (apoptosis and necrosis) for the micronucleus assay in exfoliated cells, such as karyolysis, karyorrhexis, and pyknosis. This approach is very important because cytotoxicity is a potential source of bias in the micronucleus assay. When cytotoxicity is elevated, micronucleus frequency reduces because micronucleated cells are missing as a result of cell death. In this study, the authors were not able to evaluate cytotoxicity assessment in oral mucosal cells. This certainly helps to clarify the absence of evidence for a statistically significant difference in the total number of micronuclei between groups.

We believe these commentaries are useful for better understanding this important article on the evaluation of genomic damage in pathology laboratory workers chronically exposed to formalin.
